# Studying the Degradation of Three Polymers under Different Chlorine Concentrations and Exposure Times

**DOI:** 10.3390/polym15193931

**Published:** 2023-09-29

**Authors:** Marta L. S. Barbosa, Rúben D. F. S. Costa, Francisco J. G. Silva, Susana R. Sousa, Arnaldo G. Pinto, Bruno O. Ferreira

**Affiliations:** 1 ISEP, Polytechnic of Porto, 4249-015 Porto, Portugal; martabarbosa8c@gmail.com (M.L.S.B.); rubendcosta98@gmail.com (R.D.F.S.C.); agp@isep.ipp.pt (A.G.P.); bruno3730@hotmail.com (B.O.F.); 2 INEGI—Institute of Science and Innovation in Mechanical and Industrial Engineering, 4200-465 Porto, Portugal; 3 i3S—Instituto de Investigação e Inovação em Saúde, Universidade do Porto, 4200-135 Porto, Portugal; sms@isep.ipp.pt; 4 INEB—Instituto de Engenharia Biomédica, Universidade do Porto, 4200-135 Porto, Portugal

**Keywords:** polymeric degradation, municipal facilities, chlorine action, PVC, HDPE, PP, polymers

## Abstract

Due to chlorine’s ability to kill bacteria and fungi through a chemical reaction, chlorine solutions are commonly used to clean and disinfect numerous public facilities, although these actions are also dependent to the equipment present in those facilities. Accordingly, the interest in studying its effect when in contact with different materials is obvious. This study was carried out through accelerated degradation tests and various analysis methods (optical microscope, scanning electron microscope, and tensile tests). The objective was to observe the wear presented by three polymeric materials, polyvinyl chloride (PVC), high-density polyethylene (HDPE), and polypropylene (PP), when exposed to chlorine’s action in swimming pools and drinking water treatment plants. The resulting effect depends on the chlorine content and the type of contact between the chemical agent and the material. The aim was to select the material less likely to be affected by chlorine through tests and analyses, allowing a longer component life. The use of certain more resistant polymeric materials can drastically reduce maintenance, reducing fundamental factors such as costs, the downtime of municipal facilities, and also the risk to public health. It was concluded that PVC has the most stable behaviour overall when in contact with chlorine solutions.

## 1. Introduction

Chlorine is a chemical element of the halogen group which presents the atomic number 17 and the chemical symbol “Cl” [1]. Under conditions of room temperature and normal atmospheric pressure, it is in a gaseous state with a greenish-yellow colour and a pungent smell, and is highly toxic; however, at 6.8 atmospheres and 20 °C, it changes to a liquid state [1,2]. This is a pure element that is obtained through the oxidation of hydrochloric acid or through the electrolysis of potassium or sodium chloride solutions [2].

Chlorine is commonly used for the treatment of water. It is also used as a raw material in the production of hydrogen chloride, hydrochloric acid, di-chloro-methane, and sodium hypochlorite. The latter is mainly used by the industry in the production of disinfectants and is presented in liquid form, reaching a chlorine content of 10% to 15% [3].

Since chlorine is used both for water treatment and disinfectants [4], it is associated with use in public facilities, such as municipal swimming pools or water treatment plants, for example [5]. These facilities have structures composed of different materials, among which are polymers [6]. Whether in the lane separation buoys, swimming aid buoys, water drainage structures at the pool edges, piping, or anti-slip structures at the pool access point, all these structures are usually made of polymeric materials, being constantly exposed to chlorine contact and, consequently, to its degradation [7]. Thus, it becomes particularly relevant to study the type of degradation that these materials suffer under the effect of this agent, as performed regarding other materials in contact [8]. Similar studies have been carried out on wood [9] and steel [10].

In polymeric materials, the term “degradation” is often defined as the breaking of bonds in the primary chain of polymers, with a consequent change in chemical structure and a decrease in molecular weight [11]. This degradation is evidenced by the progressive deterioration of the polymer’s mechanical properties, including its visual appearance, which leads to a decline in the material’s physical properties [12]. Polymer degradation can occur thermally, mechanically, or chemically. Degradation by contact with chlorine is classified as chemical, and affects the cohesion of the polymer structure; in short, the bonds between molecules or atoms are broken [13]. A chemical attack on polymers occurs through swelling, dissolution and, above all, chemical bond breaking (through hydrolysis, oxidation, etc.) [14,15]. It can also happen through a combination of any of these effects, thus negatively impacting the original properties and leading to the deformation of the part [16].

In general, solutions of inorganic salts and alkaline and weak acid solutions do not significantly affect plastics and elastomers. On the contrary, most organic solvents attack them, especially when hot, in a more or less intense way. This attack will be more significant the greater the similarity between the structure of the solvent and that of the polymer [17]. Polymer chain ruptures can occur due to ultraviolet light (ionising radiation), free radicals and oxidants (oxygen, ozone, and chlorine), and rupture caused by ozone [18].

Several studies have been carried out on the exposure of various polymers to environments containing chlorine. Dong et al. [19] studied the response of electroactive biofilms (EAB) to chronic chlorine exposure, concluding that EAB electroactivity could be deteriorated if intensively exposed to chlorine. Wang et al. [20] studied the integration of beta-cyclodextrin monomers through interfacial polymerisation in order to create chlorine-resistant nanofiltration membranes. Vatampour et al. [13] developed modified thin-film polyamide nanocomposite membranes for the simultaneous enhancement of antifouling and chlorine-resistance performance using a novel infinite coordination polymer (ICP), verifying that NaCl rejection improved from 97.1% to 98.8% thanks to increased surface negative charge. Zhang et al. [21] developed a membrane through forward osmosis technology produced layer-by-layer by interfacial polymerisation and grafted sulphonamide group for improving chlorine resistance and water permeability. This short review of the literature shows that the most recent studies have essentially focused on the development of polymeric membranes that are resistant to chlorine, assuming the harmful effect of this chemical product from the outset. Nevertheless, the oxidising effect on medium-density polyethylene promoted by chlorinated water has been seen in a Yu et al. [22] study, where a degradation process is verified in contact both with chlorinated water and water containing chlorine dioxide. Other than this, Majid et al. [23] assessed the types of failure that HDPE pipes suffer, but no chlorine relationship was established. Fischer et al. [24] addressed a fatigue crack growth testing of PP in chlorinated water, concluding that the resistance to fatigue decreases with increasing chlorine content, a proof of the polymer’s degradation. As for the PVC, only thermal degradation mechanisms are contemplated in the literature [25,26].

In spite of this, there are very few studies that investigate the behaviour of the most common polymeric (HDPE, PP, and PVC) components when exposed to high concentrations of chlorine, as is the case of components used in municipal water treatment plants. Hence, this work intends to present relevant results, including mass variation, microscopic analyses, and tensile resistance, after extended contact with chlorine for the purpose in view. This emphasises the novelty of this work due to the lack of previous studies under this subject.

In the municipal facilities consulted for this study, several cases of deterioration of polymeric materials were found, especially in components used in the transportation of sodium hypochlorite (NaClO). All the corrosive actions observed show that even the more expensive materials are not immune to degradation when they are not the most suitable for the purpose. In most cases, this deterioration should have been properly considered at the design stage and given certain construction details so that corrosive attacks could have been avoided or at least minimised. This study aims to identify the polymeric material with the best performance among a group of polymers previously selected based on the usual applications, giving by this way indications about what kind of polymeric components should be used in municipal facilities in locals exposed to high chlorine contents. This work also aims to extend the useful lifetime of polymer components exposed to chlorine environments.

The focus of this work is to study the degradation problems induced by the use of chlorine in municipal facilities. Therefore, the main objectives can be considered the following:

- Among the three most usual polymers used in municipal facilities, select the most suitable one regarding its further recommendation for use under severe chlorine exposure;
- Carry out accelerated degradation tests on the materials previously selected with the purpose of identifying chlorine-induced problems. To this end, the aim is to measure the operating time of certain components that are most susceptible to degradation.

## 2. Materials

This study focuses on the behaviour of polymeric materials when exposed to chlorine. These are a very versatile group, since it is possible to combine their properties to obtain certain desired characteristics. Some properties can be combined to such an extent that the polymers compete with metals in their characteristic properties, but with the advantage of greater lightness. Additionally, these materials stand out for their ease of moulding, which allows them to easily present complex shapes, as well as their elastic deformation, permitting the design of components which fit together, enabling an easy and inexpensive assembly process. These materials also have good texture, colouration, high tolerance, and precision. Additionally, the polymers are, too, resistant to degradation and have a low coefficient of friction [27].

The material selection needs to take into account a wide variety of functional factors, such as design requirements, material properties that specify these requirements, costs, and manufacturing processes. Since this paper focuses on the study of materials for contact with drinking water, a range of materials has to be considered that will not impair the quality of the water and, consequently, not pose a risk to public health or hinder the work of operators due to excessive damage to the material. With these requirements in mind, there is a huge range of materials, from which polyvinyl chloride (rigid PVC), high-density polyethylene (HDPE) and polypropylene (PP) have been selected as the final choices.

- RIGID PVC: short for polyvinyl chloride, which presents high resistance to shocks and falls, as well as low sensitivity to stress cracking. This material possesses a high stiffness compared to other thermoplastics, good chemical resistance, good adhesive bonding properties, and can be vacuum formed, welded, or thermoformed, being important in the case of breaks in pipes. It also presents good dimensional stability and easy processability [27].
- HDPE: abbreviation for high-density polyethylene, its main characteristics being non-toxicity as well as a low friction coefficient. This first property allows its contact with food and water for human consumption, reducing the risk of contamination. In addition to these already mentioned properties, HDPE is a rigid material, but light, easy to process, and low-cost, with excellent chemical resistance, and good abrasion and impact resistance [27].
- PP: short name for polypropylene, is used in cases where a higher chemical resistance is required. It also has the excellent characteristic of being able to be “welded”, an important feature in the case of pipes, as it makes it possible to reduce damage in a rupture event. Furthermore, in general, PP possesses good chemical and impact resistance and is mouldable and non-toxic, making it a very suitable material for contact with food and water for human consumption. As already mentioned in the case of HDPE, PP is easily machinable and has good resistance to regular friction [27].

Table 1 presents a summary of the main properties of the materials to be studied. Rigid PVC stands out as the material with the highest density, tensile strength, hardness and Young’s modulus, although it presents lower elongation after rupture. The remaining two materials, on the other hand, are very similar in their mechanical properties. These properties are for general types of each material, so that this study can be able to be reproduced and its results applied by other researchers.

## 3. Methodology

In order to achieve the objectives set out in the introduction, it was necessary

1. To carry out a survey of all the components that have been particularly damaged by chlorine in these facilities;
2. To identify the suitable materials to perform this study, taking into account the requirements of each product/component and the most common polymers used in that kind of municipal facilities;
3. To test those materials and to analyse the comparative results of their degradation when exposed to excessive chlorine concentrations for accelerated corrosion tests;

In order to analyse the effects of exposure to chlorine, an accelerated degradation study was carried out on three different thermoplastic polymers, obtained by injection with pellets. For their production, a horizonal injection machine was used and each one of the three polymers was produced using a different mould because of their linear shrinkage. In their injection, polymers shrink as they are cooling, so the cavity in the tool is cut to be slightly larger than the finished part. For this reason, and as the linear shrinkage of the three studied polymers is different, their moulds had to be different as well. Table 2 presents the injection conditions of the three moulds.

The PVC injection rate has to be low to avoid degradation. On the other hand, the remaining two polymers have a high injection rate so that the internal stresses can be kept at the lowest possible level.

This study was carried out using the immersion of the materials in stagnant solutions method, due to the fact that this is a simple and efficient method that achieves a high degree of degradation in a short period of time.

As with any other test, the ideal way to evaluate the results obtained is to make a comparison by changing variables, thus allowing for a better interpretation. To this end, ten samples of small dimensions (30 mm × 20 mm) and four samples of larger dimensions (140 mm × 20 mm), alongside a control sample, were produced and measured for each polymer under study. A previous weighing was performed in order to allow subsequent evaluation of weight loss/gain and the different sample dimensions were taken to permit a comparison of the influence of chlorine between sample sizes. In order to study the influence of exposure to a higher chlorine concentration, for each material five containers were prepared with a solution of different hydrous NaClO concentrations (2, 5, 25, 50, and 100), in % (*v*/*v*). To also evaluate the influence of chlorine exposure time, two small samples were immersed in each container (one for each exposure time) and, in each of the containers with 5% and 100% dilutions, two large samples were added, thus allowing half of the samples (five small and two large) to be subjected to a three-week test period, and the other half to three months. The reason behind the choice of the 5% and 100% concentrations was that drinking water and swimming pool water have a very low concentration (less than 5%), while the pipes feeding the sodium hypochlorite are in direct contact with its maximum concentration (100%), as well as reservoirs, distributors, etc. This way, both extremes could be tested in more detail, so that the real difference in the chlorine influence on the polymers was able to be assessed.

Sodium hypochlorite with an active concentration ≥13% and distilled water with a pH of 6.1 were used to perform the dilutions. The test was conducted at a temperature of approximately 21 °C, keeping it constant during the test, as well as with constant chlorine concentrations. Finally, the containers were closed and protected with a transparent film, to decrease the contact with possible impurities in the solution, but in which small holes were made to release possible gases produced by reaction of the materials and the solution.

In order to analyse the samples after the tests, the following methods were used:

- Visual inspection: Method performed using the naked eye, allowing samples to be observed before being taken from their containers, at the exact moment they are removed, and after samples’ drying and cleaning operations;
- Mass variation: Test using a Denver Instruments APX-200 Precision Analytic Balance (NY, USA), used to calculate the variation in the samples’ mass;
- Optical microscopy: Method in which an OLYMPUS model BX51M microscope (Tokyo, Japan) was used with a magnification of one hundred times (100×). In order to make it possible to observe the variation in degradation, two samples of each type of material immersed in each of the solutions with 2% and 100% sodium hypochlorite were analysed;
- Scanning electron microscopy (SEM): In this analysis, a Field Emission microscope, FEI Quanta 400 FEG (Oregon, USA) was used. This is provided with an EDAX system, allowing it to perform micro-analyses by energy dispersion. For this particular method, only the samples that suffered the greatest degradation were chosen, i.e., the ones immersed in a 100% NaClO concentration. An Energy Dispersive Spectroscopy (EDS) analysis was also conducted;
- Mechanical test (tensile tests): The tensile tests in this work were performed using a SHIMADZU (Quioto, Japan) universal tensile testing machine, Autograph AG-X 100 kN. Uniaxial tensile tests were performed on four samples of each material, immersed in different solutions and times, and also on the control sample, for comparison (a total of 15 samples). The control sample for each material was stored in dry conditions. In the first step, the samples were properly identified and marked by tracing their useful length limits (in this case, two points were marked between grips at a distance of 90 mm), so that the elongation of the material could later be verified. The sample was then clamped in the grips of the machine, and the programme for the tensile testing was run. In this evaluation, only the large samples (140 mm × 20 mm) were used. The tests were performed in a controlled room with a temperature of 25 °C, and the speed was kept constant at 20 mm/min, according to DIN EN ISO 527 [28], also ensuring a satisfactory number of points for the evaluation analysis. In the end, the evaluated samples were compared to the non-immersed sample of each material, and the rupture zone of each one was analysed. The main objective of this test was to observe the mechanical behaviour of the immersed materials for each test condition compared to the non-immersed ones.

## 4. Results and Discussion

At municipal facilities, it has been found that polymers which are exposed to certain chemicals can be corroded or dissolved. Exposure to temperatures higher than room temperature also favours materials’ deterioration. The action of chlorine accelerated the materials’ corrosion since it is a strong chemical agent, thus reacting with the material. Accordingly, in this section, the tests performed to assess the corrosion effect on the different polymers are described.

### 4.1. Visual Inspection

After two days of immersion, all the containers showed some effervescence, less at lower NaClO dilutions and more at higher concentrations. In spite of this, the samples did not change their shape, and there was no change in the colouration of the solutions.

**Figure 1:**
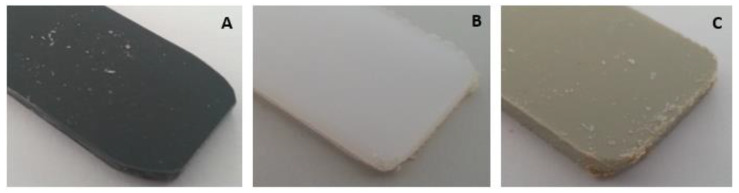
PVC (**A**), HPDE (**B**), and PP (**C**) visual inspection after 3 weeks of immersion in 100% NaClO.

After three weeks of immersion, the samples still did not change colour in either the material or the solution, and there were no degradation particles or significant changes in their size detectable by the naked eye. After the initial visual evaluation, the samples were washed with moving distilled water with the main objective of removing impurities. The samples were then kept for two days in a place at room temperature in order to dry without contact with other materials (sandpaper or similar), thus avoiding any removal of the base material. To finish this phase, a new visual evaluation was carried out, where it was possible to observe in more detail the corrosive attacks induced. In all three-week samples, after drying, degradation in the materials was still not visible to the naked eye; however, the presence of white salts on the surface of the samples was noticeable, offering some resistance to removal and, therefore, it can be seen that there was clearly an interaction between sodium hypochlorite and all polymers in study. Figure 1 shows images of these salts for one sample of each material.

After three months of immersion, the observed results were similar. There was still no change in the colouration of the solution, and the samples showed neither variations in shape nor visible degradation.

### 4.2. Mass Variation Analysis

**Figure 2:**
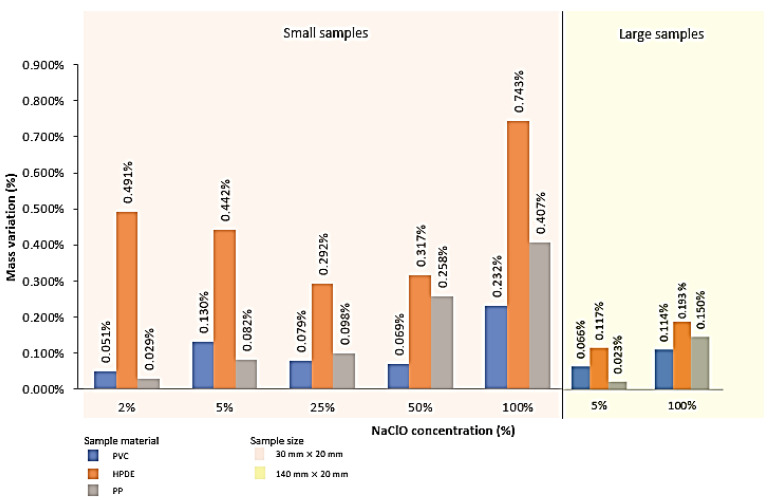
Mass variation recorded after three weeks for all samples.

**Figure 3:**
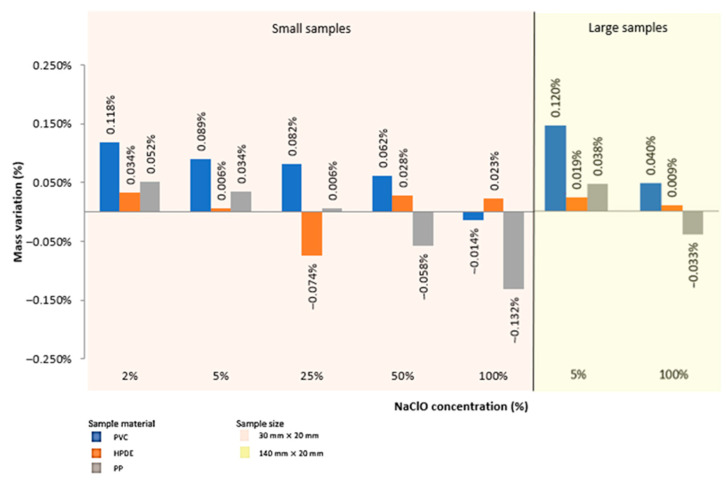
Mass variation recorded after three months for all samples.

In order to proceed with an evaluation using weight comparison, Figure 2 and Figure 3 show the mass variations obtained in each sample (first small and then large) after three weeks and three months of immersion, respectively. Additionally, Table 3 and Table 4 present the standard deviation values corresponding to each sample and also for the overall population (small and large) after three weeks and three months of immersion, respectively.

As easily observed in Figure 2 and Table 3, after three weeks of immersion, all the samples increased their mass. This increment was caused by the material’s absorption of the solution, a typical phenomenon of polymeric materials, which can cause several types of degradation. Nevertheless, the PVC and PP samples have a considerably less mass increase when compared to the HDPE. Another conclusion easily observed, found in all materials and in both the small and large samples, was a greater increase in mass in the sample that was in contact with the highest concentration of sodium hypochlorite, meaning that the absorption was greater, indicating a higher possibility of degradation.

Still, based on the results presented in Figure 2 and Table 3, exclusively analysing the PVC samples, in general, the dilutions of 2%, 25%, and 50% obtained values of mass variation close to each other, increasing sharply at the concentration of 100% of sodium hypochlorite. Analysing the HDPE samples, the values of mass variation decrease permanently until the 25% dilution of sodium hypochlorite, increasing slightly at 50%. In turn, in the PP samples, it was found that the mass variation increases constantly, being lower in the 2% dilution and higher for 100% concentration of sodium hypochlorite. Hence, it can be stated as a general trend that an increase in sodium hypochlorite concentration caused a greater increase in sample mass.

The PVC and PP samples kept some consistency until the 50% dilution, and there was a significant increase in mass in the latter, in a regular way, until the 100% concentration. It can be concluded that, in solutions containing up to 25% chlorine content, the results obtained are quite similar in the PVC and PP samples, when compared to the HDPE. On the other hand, in dilutions with 50% and 100% NaClO, the PVC reacts better to the attack caused by the solution, since it has a lower mass variation, identifying the HDPE as the one with the worst reaction to the solution since it presents a higher mass variation when compared to the other samples.

Comparing the different sample sizes, it was found that for all the materials and concentrations analysed, the larger samples had significantly less mass loss during three weeks of immersion. This suggests that smaller samples have a greater tendency to absorb solution and degradation is not perfectly clear. The larger samples either absorb less solution or start their degradation more quickly, with a simultaneous increase in mass due to the absorbed solution and a decrease due to the degradation of the material.

After three months, the results were slightly different. With the increase in the sample size, PVC and PP increased their mass, while that of HDPE decreased.

Based on Figure 3 and Table 4, by analysing the PVC samples, it can be seen that the material suffered a mass increase in almost all of them, except for the one immersed in the 100% concentration of sodium hypochlorite of smaller size, in which there was a loss of mass. Taking into account that the behaviour of this sample stands out from all the others of the same material, either taken after three weeks or after three months, it can be seen that this fact is possibly due to a strong attack created by the sodium hypochlorite over time, resulting in a loss of mass caused by the degradation of the material higher than the mass of the solution it has absorbed. It is also noticeable that the values of mass variation decrease as the concentration of sodium hypochlorite increases.

By analysing the HDPE samples, it was concluded that the material had different mass loss/gain behaviours depending on the dilution in which it was immersed. Practically all samples suffered mass increase, except the one immersed in a 25% concentration of sodium hypochlorite and, in general, the mass of the samples decreased until the 25% dilution, increasing in the 50% and keeping a variation value close to the sample immersed in 100% NaClO. Since only one of the samples showed a negative mass variation, as in PVC, it can be assumed that possibly this mass loss is due to a strong attack created by the sodium hypochlorite, which caused the degradation of the material to be higher than the mass of the fluid absorbed by it.

Analysing the PP samples, it is possible to observe that the material suffered a mass increase for lower NaClO concentrations, decreasing steadily in all samples until suffering a mass loss in the samples immersed in concentrations of 50% and 100%. As in PVC and HDPE, the increase in mass is caused by the absorption of solution by the material, which is, as already mentioned, a typical phenomenon of polymeric materials, and the loss of mass in the samples immersed in concentrations of 50% and 100% of sodium hypochlorite is possibly due to a strong attack causing degradation in the material and, consequently, leading to its mass loss. It should be noted that the sample immersed in the 25% concentration of NaClO obtained a practically null mass variation, meaning that the degradation/absorption ratio was very similar.

Comparing the two immersion times (Figure 2 and Figure 3, Table 4), it can be seen that, contrarily to the samples taken after three weeks of immersion, after three months not all samples suffered a weight increase, which denotes a greater degradation over time. In addition, it can be concluded that, in general, the samples subjected to a higher concentration of sodium hypochlorite show less mass increase. Comparing the materials among themselves, it is inferred that PVC reacts better to sodium hypochlorite in all concentrations, since there is no high mass loss when compared to the other materials. On the contrary, PP is the material that reacts worst to sodium hypochlorite.

### 4.3. Optical Microscopy Analysis

For the analysis using optical microscopy (OM) of the materials under study, magnifications of one hundred times (100×) were used on two samples of each type of material, immersed in solutions containing 2% and 100% sodium hypochlorite.

**Figure 4:**
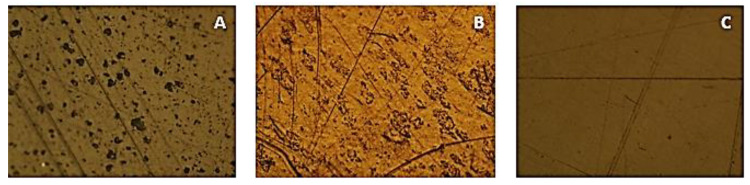
OM images of the PVC (**A**), HPDE (**B**), and PP (**C**) samples not immersed in NaClO concentration at 100× magnification.

Before being submitted to the immersion test, the samples were in an atmospheric environment, thus being exposed to possible contamination or scratches, caused by contact with other materials. In Figure 4, a sample of each material before being immersed in NaClO can be observed. In these specimens, small black spots are visible, possibly representing the presence of elements added to the base material, and some lines, caused by the cutting of the samples. The non-cleaning or extra care of the materials’ surface was intended to simulate the state in which materials in a municipal facility may be found.

**Figure 5:**
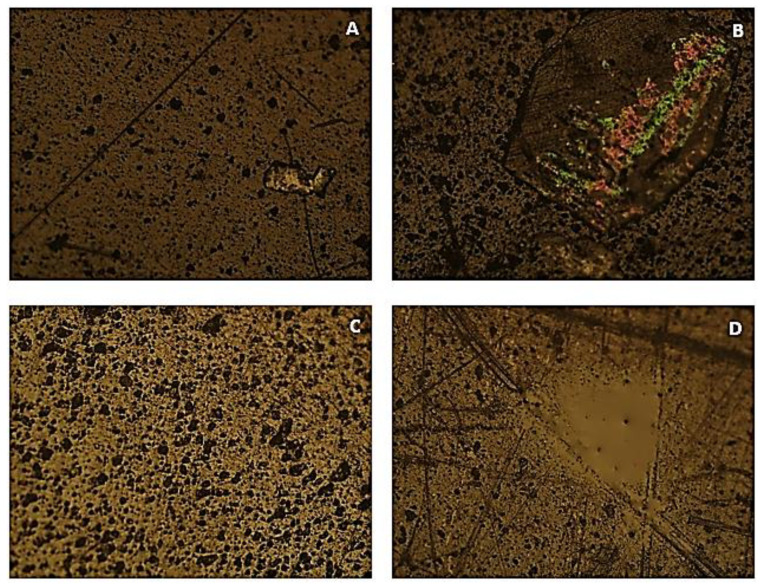
OM images of the PVC samples submitted to accelerated degradation at 100× magnification ((**A**)—sample submitted to 2% NaClO concentration for three weeks; (**B**)—sample submitted to 100% NaClO concentration for three weeks; (**C**)—sample submitted to 2% NaClO concentration for three months; (**D**)—sample submitted to 100% NaClO concentration for three months).

Figure 5 presents the microscopic images obtained of the selected PVC material samples.

In images A and B of Figure 5, light areas of irregular shape and larger dimensions can be observed, which may correspond to regions of attack with greater intensity. Multiple black spots are also visible, representative of material contamination, either by elements that interacted with it or by calcium reinforcements. Comparing images A and B, it can be concluded that these black spots increased with the increase in chlorine concentration. Image B presents a pertinent characteristic, which is the existence of a region with green and pink colouration, possibly due to the reflection of the microscope light in a region with a higher concentration of salts, clearly indicating the existence of a higher degradation. Images C and D also show dark circles and other dark regions of larger dimensions and irregular shape that correspond to areas more attacked by the solution. An interesting fact that can be verified in the sample immersed in the 100% NaClO concentration (D) is the presence of a light and an irregularly shaped area, located in the central region of the image, which possibly represents the presence of fluid traces.

Comparing the samples taken after three months of immersion with the ones after three weeks, one can conclude that the same form of degradation is observed, but with greater intensity, observing a greater quantity of black spots, as well as their greater size and regions with clearly greater degradation.

Regarding these observations, it is possible to state that samples immersed in the 100% NaClO concentration present a higher level of degradation than the samples immersed in 2% dilution. The latter, in turn, presents a higher degradation than the control sample without contact with NaClO (In Figure 4A) both after three weeks and three months of immersion. Hence, the degradation of this material is influenced by both the concentration of sodium hypochlorite and the duration of the test. Thus, the analysis using optical microscopy confirmed the results previously observed through visual inspection and confirmed by the mass variation analysis.

**Figure 6:**
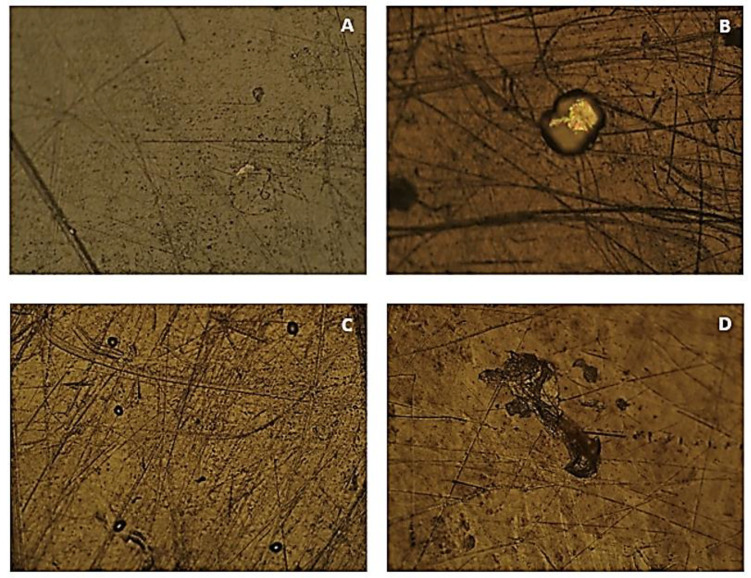
OM images of the HDPE samples submitted to accelerated degradation at 100× magnification ((**A**)—sample submitted to a concentration of 2% NaClO for three weeks; (**B**)—sample submitted to a concentration of 100% NaClO for three weeks; (**C**)—sample submitted to a concentration of 2% NaClO for three months; (**D**)—sample submitted to a concentration of 100% NaClO for three months).

Figure 6 shows surface images of the HDPE samples.

As can be seen in the images of Figure 6, all the analysed samples still present the same handling marks, and small circular spots of contamination are visible in the material. In image A, it is possible to observe a clear irregularly shaped and larger area, which was not visible before the accelerated degradation and may correspond to a region of degradation with greater intensity. In image B, there is a large area with light colouring and irregular shape and, as in the PVC sample submitted to the same concentration and time period, a region with pink and greenish colouring is also visible, possibly corresponding to the accumulation of salts and consequent further degradation. The sample in image C remained immersed for a longer period than the one in image A, and presents a larger number of dark-coloured spots, indicating greater degradation. In turn, image D, submitted to a higher concentration, shows more intense and larger dark and irregular areas. With these observations, it can be reconfirmed that in HDPE there is a greater degradation as the concentration of NaClO and the exposure time increase.

**Figure 7:**
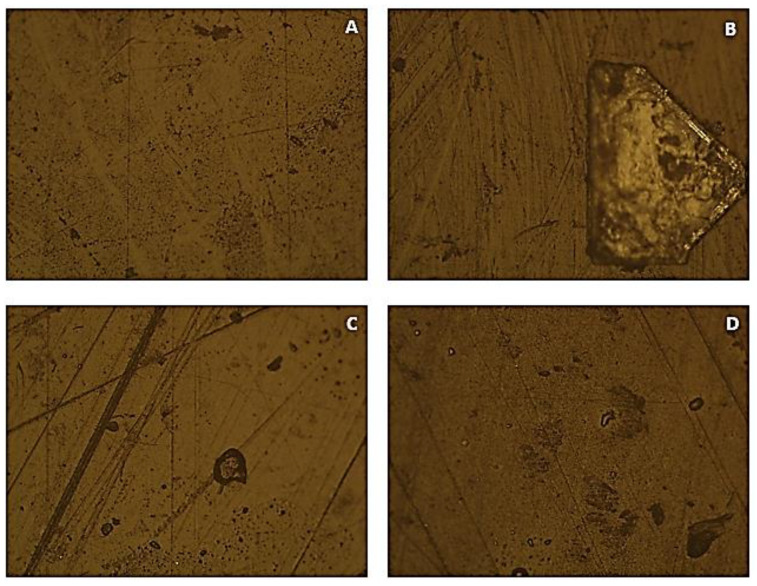
OM images of the PP samples submitted to accelerated degradation with 100× magnification ((**A**)—sample submitted to a concentration of 2% NaClO for three weeks; (**B**)—sample submitted to a concentration of 100% NaClO for three weeks; (**C**)—sample submitted to a concentration of 2% NaClO for three months; (**D**)—sample submitted to a concentration of 100% NaClO for three months).

Figure 7 presents the results obtained by optical microscopy for the PP samples.

By observing Figure 7, all the images show some dark areas, irregularly shaped and of considerable size, which may correspond to regions degraded with some intensity, some lines typical of the processing and handling, and also some lighter areas that possibly represent points of more significant degradation of the material. In image B, a large area with a different colouring of the base material can be observed. This irregular and large area clearly corresponds to a zone of greater degradation of the material. It can then be stated that the sample immersed in 100% NaClO presents a higher level of degradation when compared to the sample immersed in the 2% concentration. In image C, both a large region of dark edges and a light interior and several small dark regions are evident. Comparing this image to the one that was immersed for less time (A), the existence of increasingly pronounced dark regions is highlighted, thus evidencing greater degradation. In image D, a greater number of areas of different colouring from the base material can be seen when compared to sample C, with some areas being light-coloured and others dark, with an irregular shape and larger dimensions. It can then be stated that the sample immersed in 100% NaClO for three months presents a higher state of degradation than the 2% concentration, which is in line with that recorded in the other conducted tests. It can be concluded that the degradation of this material is also influenced by the concentration of sodium hypochlorite, as well as by the duration of the test.

Analysing the three materials together, it is possible to observe a particularly interesting result, which is the fact that the images with the same NaClO concentration and immersion time present similarities among them. As can be seen in Figure 5, Figure 6 and Figure 7, in the A images (2% for three weeks), there are clear areas, all the B images (100% for three weeks) present salt lodgement, every one of the C images (2% for three months) show many small black coloured spots, and, finally, in the D images (100% for three months) there are always very pronounced spots of degradation. It can also be confirmed that the PVC demonstrated a better behaviour to the chlorine degradation and the PP was the most affected material.

### 4.4. Scanning Electron Microscopy Analysis

**Figure 8:**
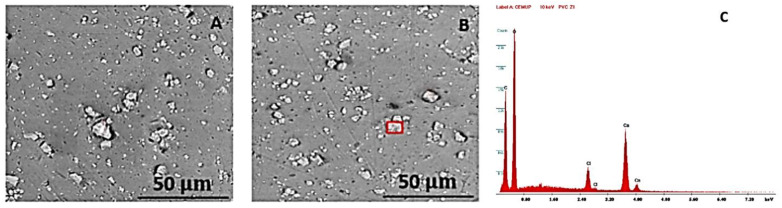
SEM images of the PVC sample with 200× magnification after three weeks (**A**) and three months (**B**) of immersion. (**C**) EDS graph of the red zone in (**B**).

**Figure A1:**
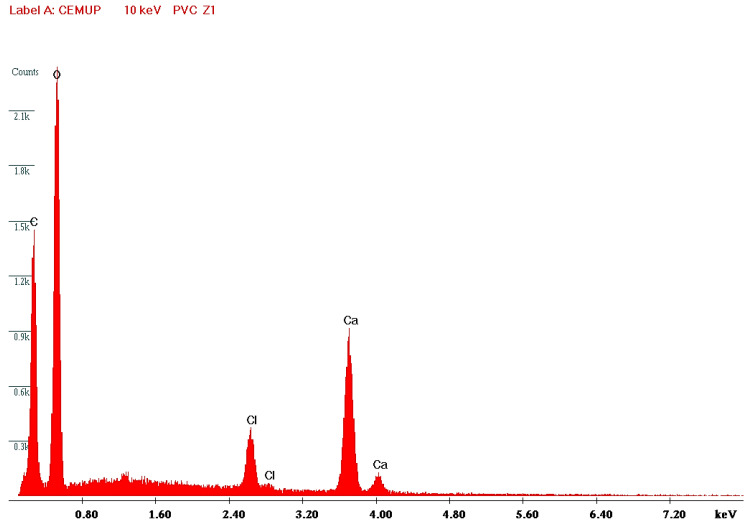
EDS of the red zone in the second image of the PVC sample.

After the analysis with the optical microscope, a SEM analysis was also performed, always with a 2000x magnification, in order to analyse the samples’ surface in more detail, as a way of better evaluating the degradation suffered by the polymers. An EDS analysis was also conducted on the PVC and HDPE samples to understand how their chemical composition was affected by the NaClO. The EDS analysis in the PP could not be performed due to contamination of the sample. Figure 8 shows the PVC sample in the SEM, firstly after three weeks (A) and then after three months (B). Image C presents the EDS graph for the red zone in (B) and can be consulted in Figure A1 of the Appendix A.

In both SEM images (A and B), there are no visible signs of degradation due to the chlorine. After three months, the microscopic view is identical to the one after three weeks, with only some smooth scratches barely visible on its surface, which means it has presented a good degradation resistance. The dark areas constitute impurities in the sample’s surface, and the clear crystals are a reinforcement of the polymer, composed mainly of calcium, as can be seen in image C. High values of oxygen and carbon are also visible, meaning an initial degradation of the polymeric material. This is in line with the optical microscope analysis, where this material did not show a high level of degradation.

**Figure 9:**
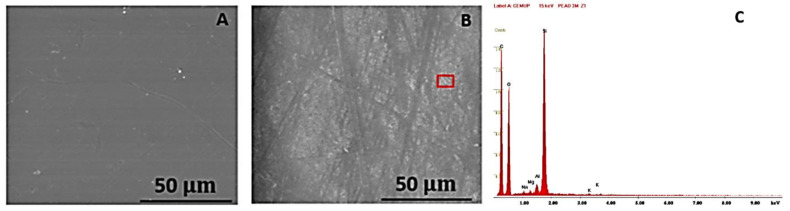
SEM images of the HDPE sample with a 2000× magnification after three weeks (**A**) and three months (**B**) of immersion. (**C**) EDS graph of the red zone in (**B**).

**Figure A2:**
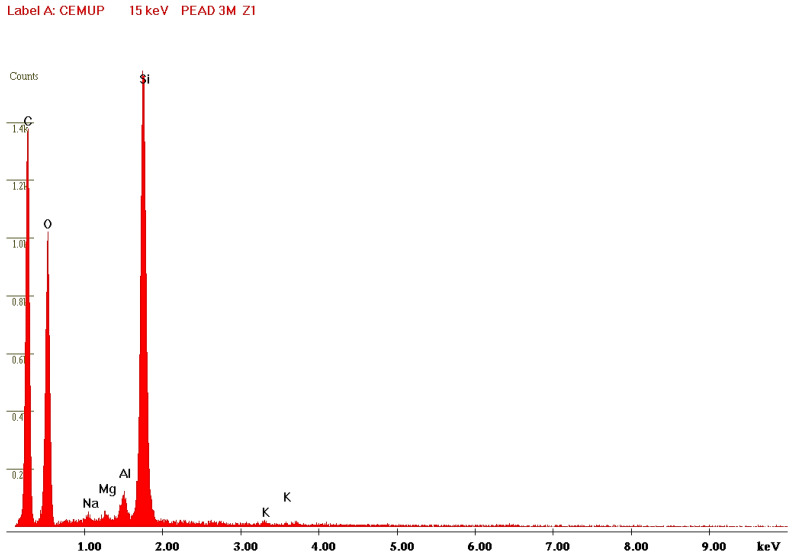
EDS of the red zone in the second image of the HDPE sample.

Figure 9 represents the SEM images of the HDPE after three weeks (A) and three months (B) of immersion. In image C, it is possible to observe the EDS graph of the red square in the second image, also seen in more detail in Figure A2 of the Appendix A.

In opposition to what was registered in PVC, although in image A only some small white spots are visible, due to the salt grains released by the solution, in image B all the sample is covered in a clear cloak, a generalised degradation caused by the NaClO, which, although it is not too deep, done more damage than in the former material. Some lines across the sample are also visible, possibly made by the handling of the sample during its processing. Image C shows a great amount of silicon, a superficial impurity, but also high values of carbon and oxygen, meaning an increasing degradation.

**Figure 10:**
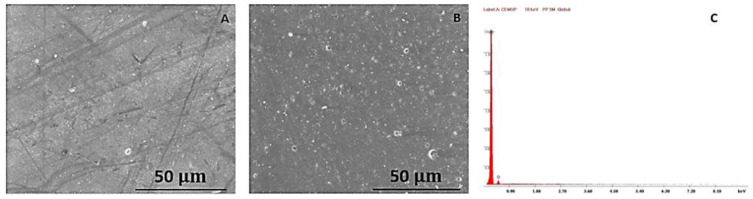
SEM images of the PP sample with a 200× magnification after three weeks (**A**) and three months (**B**) of immersion.

**Figure A3:**
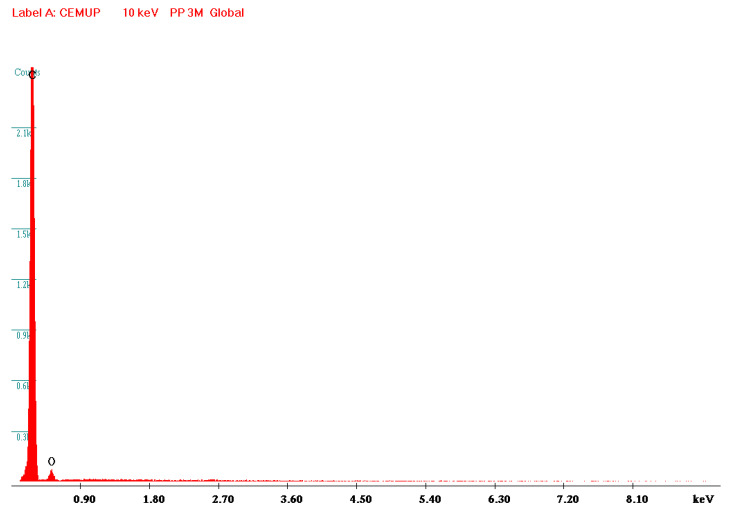
EDS of the second image of the PP sample.

Finally, in Figure 10, the PP samples are observable using SEM after three weeks (A) and after three months (B) of immersion. In image C, the EDS graph relative to the entire scanned area of the PP sample after three months in the SEM is presented, but it may be observed in more detail in larger scale in Figure A3 of the Appendix A

This material is clearly the most degraded of the three, reinforcing the conclusions taken from the previous analyses, with some white spots already visible after three weeks (A), representative of the degradation beginning to arise on the sample. Some risks are also present due to the handling of the material in the processing phase. In image B, the consequences of the three months of immersion are noticeable, as the whole sample shows a variety of white spots, varying in size and shape, which denote its worse capacity for resisting to the chlorine immersion. Image C, although barely visible, has a peak of carbon and also a small peak of oxygen, denoting its first degradation signs. Nevertheless, not many chemical elements show in the EDS, because, contrarily to the other two materials, this graph is a global one, so it just shows the substances present in the overall area of the sample. Still, it is important to complement the data present in the remaining images.

### 4.5. Tensile Test

The facts verified in this analysis can be influenced by not pre-treating the samples, which can occasionally influence resistance. Nevertheless, this study aims to analyse the behaviour of the materials under service conditions in contact with chlorine, also without any kind of treatment until their total degradation.

**Figure 11:**
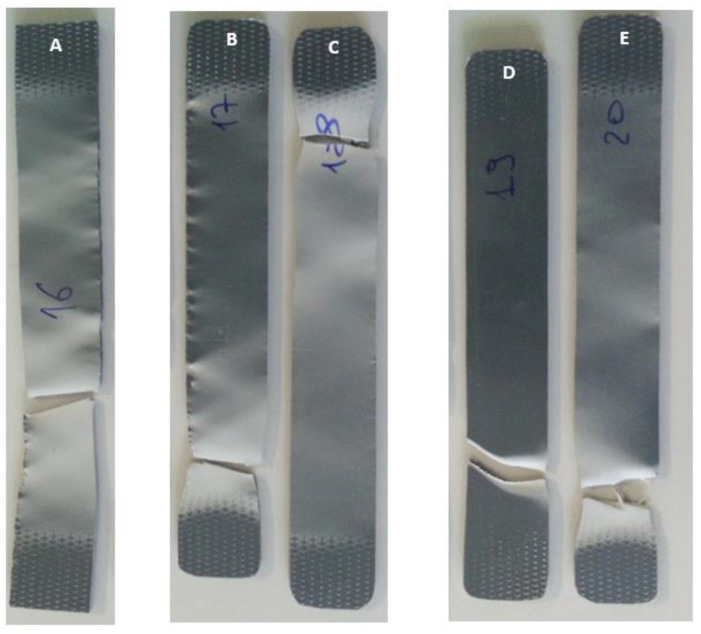
Tensile tests of PVC samples ((A)—control sample; (B)—sample immersed for three weeks in 5% NaClO; (C)—sample immersed for three weeks in 100% NaClO; (D)—sample immersed for three months in 5% NaClO; (E)—sample immersed for three months in 100% NaClO).

Figure 11 shows the PVC samples submitted to the tensile test. It is possible to observe that the results obtained were distinct, both in terms of rupture and elongation.

By analysing Figure 11, it can be seen that both the samples immersed for three weeks, in 5% (B) and 100% (C) NaClO, and those immersed for three months, also in 5% (D) and 100% (E) NaClO, collapsed in approximately the same zone, more precisely near the clamping zone of the testing machine’s claws, but still into the useful length region. On the other hand, the control sample broke near the central zone. In addition, there were regions of whitish colouration throughout the entire working length of the control specimen, while in the other specimens this same whitish colouration can be observed only near the rupture zone, keeping a colouration similar to the original in the remaining working length of the specimen. In all samples, the fracture is practically perpendicular to the direction in which the stress was applied, and the strain zone was relatively unpronounced, a phenomenon characterised by the reduction in cross-sectional area, very common in polymers. Analysing in detail the rupture zone of the samples used in the tensile tests, a greater degradation aspect is observed with the increment of the NaClO concentration. In this material, the fracture aspect is smooth in the control sample, and fibrous in the samples immersed in the solution, being more pronounced the higher the concentration of NaClO used. Comparing the samples in terms of immersion time (B with D and C with E), samples D and E show more signs of embrittlement, as evidenced by a transverse crack in D and by multiple fibres in E. Therefore, the analysis of these images confirms once again what was already verified by the other tests, that the increase in concentration and exposure time to NaClO leads to a higher degradation of the PVC.

**Figure 12:**
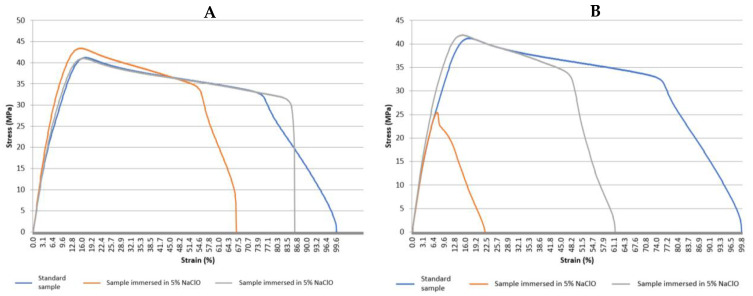
Stress–strain curve graphs for the PVC tensile tests for the control sample and the samples immersed in 5% NaClO and 100% NaClO for three weeks (**A**) and three months (**B**).

Figure 12 shows the graphs with the stress–strain curves of the PVC tensile tests for the control sample and the samples immersed in 5% NaClO and 100% NaClO for three weeks (A) and three months (B), and Table 5 shows the values reached by PVC at the point of maximum load during the tensile test on the samples analysed, with the standard deviation for each sample and also for the overall population.

In Table 5, the sample immersed in 5% NaClO for three months was rejected from the calculations due to having had a problem, as the values are clearly completely different from all the remaining ones.

Observing Figure 12 and the values in Table 5, the visual analysis performed in a first stage is confirmed, in which it is possible to verify that the mechanical properties of the samples immersed in 5% and 100% NaClO were affected by the degradation caused by the NaClO solution. Both after three weeks and three months, they showed a significantly different behaviour compared to the control one. With the exception of the curve referring to the 5% NaClO sample immersion for three months, it can be concluded that there is a characteristically plastic deformation in the samples in the fracture region, which is typical of a ductile fracture. Once again, excepting the 5% sample for three months, the samples show similar behaviours in the elastic domain, with different behaviours only observed in the material’s plastic and rupture domains. It can also be stated that, with the same exception, the samples submitted to the test demonstrated similar maximum strength and elongation values. In general, it can be concluded that the behaviour of the samples was similar in the elastic domain, but the control sample showed a greater deformation in the plastic zone. Focusing on the samples immersed for three months, it can be verified that after reaching the maximum load, there is a significantly higher elongation in the control sample compared to the sample immersed in 100% NaClO. This fact can be explained by the degradation suffered by the materials immersed in NaClO, reducing their plastic properties as the NaClO content increases. This phenomenon is demonstrated in this case by an embrittlement of the material, while the control sample continues to deform plastically in the rupture phase, presenting, therefore, a higher elongation.

It should be noted that, except for the one immersed in 5% NaClO for three months, all the samples had the expected behaviour, since the ones immersed in NaClO solution showed greater signs of degradation compared to the control one, which is reflected in the values obtained by the tensile test. This can be explained by the fact that the control sample obviously did not present degradation in the specimen length area, unlike the samples immersed in 5% and 100% NaClO.

**Figure 13:**
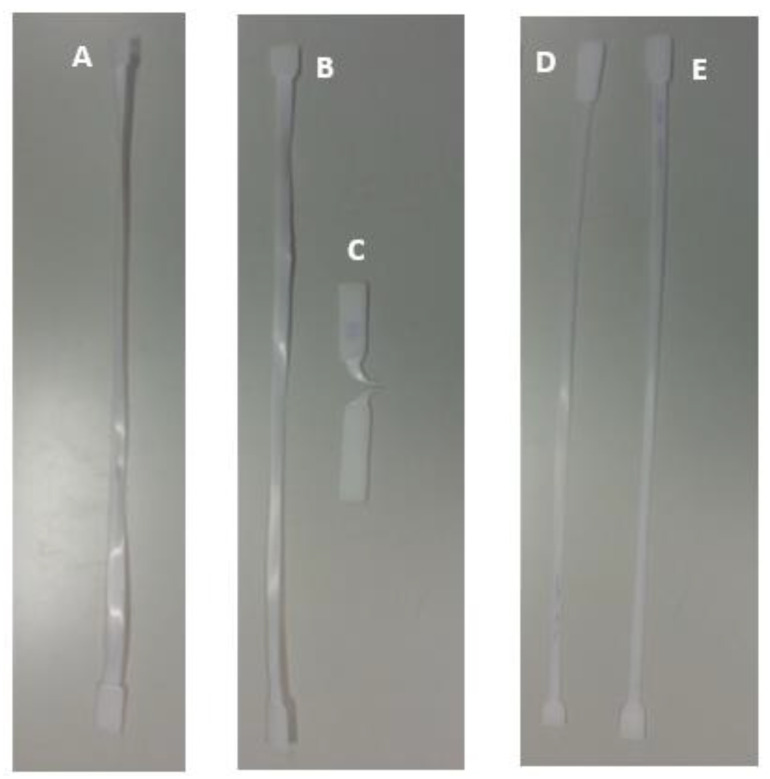
Tensile test HDPE samples ((A)—control sample; (B)—sample immersed for three weeks in 5% NaClO; (C)—sample immersed for three weeks in 100% NaClO; (D)—sample immersed for three months in 5% NaClO; (E)—sample immersed for three months in 100% NaClO).

Figure 13 shows the HDPE samples that were subjected to tensile testing.

Observing Figure 13, it is possible to verify that, with the exception of the sample immersed in 100% NaClO for three weeks (C), all the samples presented a similar behaviour, showing high elongation, but without rupture. On the contrary, the referred sample suffered a rupture near the central zone. The latter have fractured without significant elongation when compared to the other samples. Visually, it was not possible to observe in the samples any sign of degradation caused by the attack of the NaClO solution. All the remaining specimens presented high deformation, without rupture, and the test was interrupted when the maximum limit of the machine stroke was reached. It is also observed that the deformation of the samples is very homogeneous, and they present a uniform reduction in dimensions in the tensile area, which means that they possess a high resistance to developing a bottleneck region. In addition, there is a clear decrease in cross-section at each end of the sample due to the high plastic deformation of this material.

**Figure 14:**
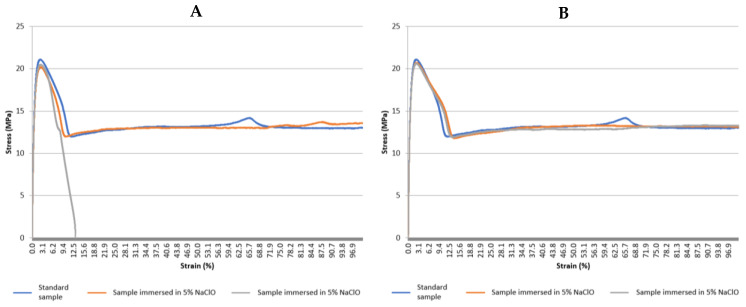
Stress–strain curve graphs for the HPDE tensile tests for the control sample and the samples immersed in 5% NaClO and 100% NaClO for three weeks (**A**) and three months (**B**).

Figure 14 shows the graphs with the stress–strain curves of the HDPE tensile tests for the control sample and the samples immersed in 5% and 100% NaClO for three weeks (A) and three months (B). Moreover, Table 6 shows the values reached by HDPE at the point of maximum load during the tensile test on the analysed samples, with the standard deviation for each sample and also for the overall population. The sample immersed in 100% NaClO for three weeks has a distinct curve, without any results which can be taken from it, for the reason already explained in the samples’ analysis.

By observing Figure 14, the visual analysis performed in the first stage is confirmed, in which it is possible to verify that, with the exception of the sample immersed in 100% NaClO for three weeks, all the others present a similar mechanical performance. The remaining samples show a characteristically plastic deformation without rupture, with an elongation of approximately 500 mm, corresponding to the stroke limit of the testing machine. Additionally, Table 6 confirms the analysis performed through the graph in Figure 11, allowing the observation that all samples presented similar maximum load values. There was also a similar elongation until the maximum strength was reached, with rupture occurring only in the sample immersed in 100% NaClO for three weeks. It should be noted that the samples behaved as expected since the high plastic deformation is one of the main characteristics of HDPE, which resists quite well to damage by chemical agents such as NaClO. Therefore, it was not possible to observe signs of the degradation of the samples immersed in NaClO when compared to the control one, as reflected in the values obtained in the tensile test.

**Figure 15:**
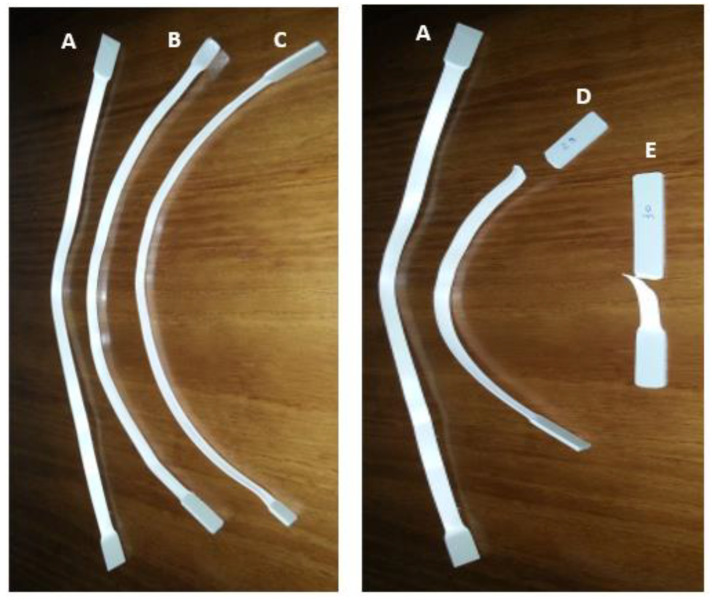
Tensile test of PP samples ((A)—control sample; (B)—sample immersed for three weeks in 5% NaClO; (C)—sample immersed for three weeks in 100% NaClO; (D)—sample immersed for three months in 5% NaClO; (E)—sample immersed for three months in 100% NaClO).

Figure 15 shows the PP samples subjected to the tensile test.

With the analysis of Figure 15, it is possible to verify that the control sample (A) and the samples immersed in NaClO for three weeks (B and C) presented a similar behaviour, in which a significant elongation can be observed, without the occurrence of rupture; however, it is not possible to visually observe any signs of degradation caused by the attack of the NaClO solution. In the samples subjected to chlorine for three months (D and E), it is possible to verify that they presented rupture, but a high elongation can be observed in the sample immersed in 5% NaClO (D). The sample immersed in 100% NaClO (E) broke in the central zone, while the sample immersed in 5% NaClO (D) cracked near one of its extremities, being, however, in the test valid region. Visually, it can be stated that the sample immersed in 5% NaClO presents a behaviour with characteristics contained between the observed in the control sample and in the one immersed in 100% NaClO. This was expected, since, theoretically, the strength of the NaClO action in the sample immersed in 5% is between the values presented by the control sample and the one immersed in 100% NaClO. Nevertheless, observing the rupture surface of the samples, it is not possible to clearly observe any sign of degradation caused by NaClO solution action. The elastic deformation zones of the control sample and the samples immersed for three weeks are close to each other, showing a similar deformation behaviour, i.e., a large deformation, without rupture. As in the HDPE samples, the deformation of the samples is homogeneous, presenting a uniform decrease in the tensile useful area with a clear decrease in cross-section at each end of the sample. Regarding the samples immersed for three months, the specimen immersed in 5% NaClO presents a high plastic deformation, with a clear decrease in cross-section at each end of the specimen (similar to the control sample), but occurring rupture (similar to the specimen immersed in 100% NaClO). The fracture was perpendicular to the direction of the tensile load, meaning the material was fragile; however, the sample presented a considerably large elongation, and thus it is possible to conclude that the plasticity of the material was affected by the NaClO solution action. In the sample immersed in 100% NaClO, plastic deformation in the rupture zone is verified, as well as a clear decrease in the cross-section at one end of the sample. With this observation, it can be concluded that the sample immersed in 100% was much more affected by the NaClO than the others, as expected, translating into a loss of properties in the plastic domain.

**Figure 16:**
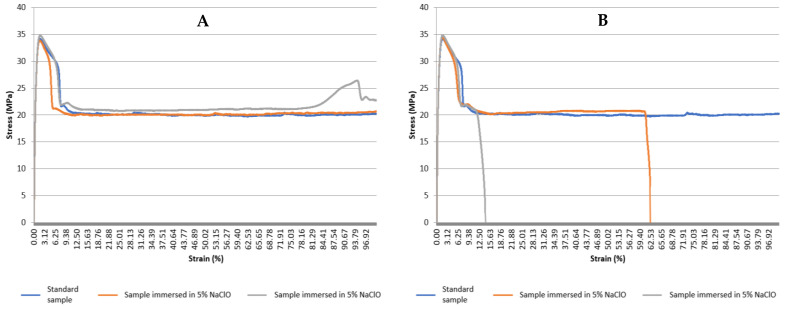
Stress–strain curve graphs for the PP tensile tests for the control and the samples immersed in 5% NaClO and 100% NaClO for three weeks (**A**) and three months (**B**).

Figure 16 shows the graphs with the stress–strain curves of the PP tensile tests for the control sample and the samples immersed in 5% NaClO and 100% NaClO for three weeks (A) and three months (B). Moreover, Table 7 shows the values reached by the same material at the point of maximum load during the tensile test on the analysed samples, with the standard deviation for each sample and also for the overall population.

Observing Figure 16, the visual analysis performed in the first stage is confirmed. The mechanical properties of both the control sample and the ones immersed for three weeks showed similar behaviour, presenting a characteristically plastic deformation without rupture, with an elongation of approximately 500 mm, which corresponds to the stroke limit of the tensile testing machine, a homogeneous deformation. It was also confirmed that the samples immersed for three months showed rupture. The specimen immersed in 5% NaClO showed a considerably higher elongation; however, there were signs of degradation through the rupture that occurred in the material and, by comparison with the other samples, the samples presented considerable indices of fragility. The sample immersed in 100% NaClO showed little plastic deformation, and it is possible to conclude that the material suffered high degradation, since the results obtained were quite different from the other samples, demonstrating the fragility of the material in the rupture zone due to the degradation.

In general, it was found that all samples presented similar behaviour in the elastic domain, as at the beginning of the test the curves are all overlapped, with different behaviour only in the plastic domain of the material, this difference being visible when exposed to NaClO solution for three months. These samples presented the expected behaviour, with a higher plastic deformation in the sample immersed in 5% NaClO when compared to the sample immersed in 100% NaClO, and a subsequent rupture in both of them. The values in Table 7 prove the validity of the analysis performed through the graph in the elastic domain, and it can be verified that all samples presented similar maximum loads and elongations. These facts lead to the conclusion that the NaClO solution degrades the PP material in such a way that its mechanical properties are altered. This alteration is higher the longer the exposure time and the concentration of the solution.

Comparing the three materials, PVC was the one that presented the best performance in this test, because although all the specimens have fractured, it was the material that supported a higher maximum load and presented the lowest deformation.

## 5. Conclusions

The study of the degradation caused by chlorine, as well as the search for alternative materials, was conducted with cost-accessible materials, as it is possible for any installed material to be replaced with components made of these polymers, depending on the type of accessories or components needed and their function in service, etc. The evaluations were performed without any preparation, with the objective of studying materials subjected to direct contact with various solutions of NaClO (2%, 5%, 25%, 50%, and 100%, with a special focus on the 5% and 100%, as extremes), keeping the usual conditions found in real situations of use. Focusing mainly on the materials that can be used as alternatives in most of the components and accessories of the targeted installations, the materials chosen for the study were PVC, HDPE, and PP.

Polyvinyl chloride (PVC) was the polymer that showed the most stable behaviour for any immersion time and NaClO concentration, in spite of losing elongation both for 3 weeks and 3 months. Nevertheless, in all the other tests, it demonstrates the best results, supports a higher maximum load, and has a lower deformation.

Polypropylene (PP) shows the worst results in almost all the tests. For shorter immersions (three weeks), there is no significant change, both in terms of mechanical strength and elongation, regardless of the concentration. For three months, it loses more ductility the higher the NaClO concentration, and in the remaining tests a high level of degradation is observed.

Finally, high-density polyethylene (HDPE) was the polymer which presented the worst behaviour in terms of mass loss due to degradation. A great evolution of degradation is seen, and although it keeps its mechanical strength, it loses ductility after 100% NaClO and 3 weeks of immersion.

It was observed that the degradation of polymers, although not very easily visible, increases with the period of time of exposure to a corrosive medium, as well as with the increase in the content of the corrosive agent used, which manifests itself by swelling at an early stage, and loss of mass later on, as well as loss of elongation capacity.

In conclusion, none of the three tested materials has a perfect result in degradation resistance; however, the one which showed the most stable results, in addition to supporting a higher load and having lower deformation was the polyvinyl chloride (PVC), making it a good choice for application in municipal facilities, particularly for swimming pool equipment and drinking water treatment plants. Nevertheless, in the final choice of this material for the referred applications, its processing could make it more expensive than the other materials tested, depending on the shape and function of the component in whose production it is used.

## Figures and Tables

**Table 1:** Summary of the main physical and mechanical properties of the materials to be studied.

Material	Rigid PVC	HDPE	PP
Density (g/cm^3^)	1.44	0.95	0.91
Tensile strength (MPa)	58	28	33
Elongation after rupture (%)	4	8	8
Hardness (R)	93	65	86
Young’s modulus (GPa)	3.3	1.1	1.7

**Table 2:** Injection conditions of the three materials to be studied.

Material	Rigid PVC	HDPE	PP
Linear shrinkage (%)	0.4	2.3	1.5
Injection temperature (°C)	190	230	250
Cooling temperature (°C)	45	60	50
Injection pressure (MPa)	100	105	180
Injection rate	Low	High	High

**Table 3:** Standard deviation values for the three materials in the mass variation test after three weeks.

Type of Sample		Small Samples						Large Samples		
NaClO Concentration		2%	5%	25%	50%	100%	Average	5%	100%	Average
PVC	Mass variation	0.051	0.130	0.079	0.069	0.232	0.112	0.066	0.114	0.090
	Deviation	0.061	0.018	0.033	0.043	0.120	0.073	0.024	0.024	0.034
HDPE	Mass variation	0.491	0.442	0.292	0.317	0.743	0.457	0.117	0.193	0.155
	Deviation	0.034	0.015	0.165	0.140	0.286	0.180	0.038	0.038	0.054
PP	Mass variation	0.029	0.082	0.098	0.258	0.407	0.175	0.023	0.150	0.087
	Deviation	0.146	0.093	0.077	0.083	0.232	0.155	0.064	0.064	0.090

**Table 4:** Standard deviation values for the three materials in the mass variation test after three months.

Type of Sample		Small Samples						Large Samples		
NaClO Concentration		2%	5%	25%	50%	100%	Average	5%	100%	Average
PVC	Mass variation	0.118	0.089	0.082	0.062	−0.014	0.067	0.120	0.040	0.080
	Deviation	0.051	0.022	0.015	0.005	0.081	0.050	0.040	0.040	0.047
HDPE	Mass variation	0.034	0.006	−0.074	0.028	0.023	0.003	0.019	0.009	0.014
	Deviation	0.031	0.003	0.077	0.025	0.020	0.045	0.005	0.005	0.007
PP	Mass variation	0.052	0.034	0.006	−0.058	−0.132	−0.020	0.038	−0.033	0.003
	Deviation	0.072	0.054	0.026	0.038	0.112	0.075	0.036	0.036	0.050

**Table 5:** Values reached by the PVC at the point of maximum load during the tensile test on the samples analysed.

Immersion Time	Sample	F_max_ (N)	Standard Deviation	σ_max_ (MPa)	Standard Deviation	Displacement (mm)	Standard Deviation
	Control	2473.84	40.17	41.20	0.69	4.64	0.00
Three weeks	5% NaClO	2605.49	91.48	43.42	1.53	4.28	0.36
	100% NaClO	2462.78	51.23	41.05	0.84	5.51	0.87
Three months	5% NaClO	1525.91	-	25.43	-	2.04	-
	100% NaClO	2513.93	0.08	41.90	0.01	4.14	0.50
	Average (excluding 5% NaClO sample)	2514.01	64.82	41.89	1.08	4.64	0.62

**Table 6:** Values reached by the HPDE at the point of maximum load during the tensile test on the samples analysed.

Immersion Time	Sample	F_max_ (N)	Standard Deviation	σ_max_ (MPa)	Standard Deviation	Displacement (mm)	Standard Deviation
	Control	1266.21	28.60	21.10	0.52	11.78	0.45
Three weeks	5% NaClO	1211.96	25.65	20.20	0.38	12.21	0.02
	100% NaClO	1229.59	8.02	20.49	0.09	12.88	0.65
Three months	5% NaClO	1243.64	6.03	20.49	0.09	12.01	0.22
	100% NaClO	1236.65	0.96	20.61	0.03	12.28	0.05
	Average	1237.61	19.86	20.58	0.33	12.23	0.41

**Table 7:** Values reached by the PP at the point of maximum load during the tensile test on the samples analysed.

Immersion Time	Sample	F_max_ (N)	Standard Deviation	σ_max_ (MPa)	Standard Deviation	Displacement (mm)	Standard Deviation
	Control	2051.51	14.23	34.19	0.24	8.64	0.07
Three weeks	5% NaClO	2032.63	33.11	33.88	0.55	8.48	0.09
	100% NaClO	2088.72	22.98	34.81	0.38	8.78	0.21
Three months	5% NaClO	2065.77	0.03	34.43	0.00	8.18	0.39
	100% NaClO	2090.07	24.33	34.83	0.40	8.78	0.21
	Average	2065.74	24.59	34.43	0.41	8.57	0.25

## Data Availability

Not applicable.
